# Atrial Fibrillation and Revascularization Procedures: Clinical and Prognostic Significance. Incidence, Predictors, Treatment, and Long-Term Outcome

**Published:** 2007-02-14

**Authors:** Paolo Terranova, Francesca Carletti, Paolo Valli, Simonetta Dell'Orto, Greco Enrico Maria, Peppino Terranova

**Affiliations:** 1 Divisione e Cattedra di Cardiologia, Dipartimento di Medicina, Chirurgia e Odontoiatria, Azienda Ospedaliera "S. Paolo", University of Milan, Italy; 2 Unita Operativa di Cardiologia, Presidio Ospedaliero "Causa Pia Uboldo", Cernusco Sul Naviglio, Azienda Ospedaliera di Melegnano, Milano; 3 Divisione di Cardiologia, Azienda Ospedaliera "Luigi Sacco" - Polo Universitario, Istituto di Scienze Cliniche LITA, University of Milan, Italy

Manuscript retracted by first author with apologies for erroneous submission as it was quite similar to another article published by another author in a workshop proceedings. The author mentioned that it was a non voluntary mistake and requested immediate erasal of the paper. The author has also informed that the co-authors had no role in this error.

